# Supplementary material for: Pregnancy and Caffeine Metabolism: Updated Insights and Implications for Maternal–Fetal Health
Source: Nutrients. 2025 Oct 8;17(19):3173. doi: 10.3390/nu17193173 (PMC12526311; doi:10.3390/nu17193173)
Supplement: Supplementary file 1 [file nutrients-17-03173-s001.zip › nutrients-3903654-supplementary.pdf]

Supplementary Table S1. The summary of (A) clinical trials and (B) meta-analyses on the effects of caffeine during pregnancy on maternal and offspring outcomes 1

Study, year	Study design and comparator	Number of participants	Exposure assessment	Condition/ Population	Follow-up duration (if applicable)	Endpoints/Measures	Outcomes/ Key Findings	Limitations
						**A. CLINICAL STUDIES**		
**Developmental Delays in Children**								
Nishihara, S., et al. (2022) [1]	Prospective birth cohort study	87,106	Self-administered FFQ	Children at 6 and 12 months of age	6- and 12-month assessment	• Prenatal caffeine exposure<br>• Communication of children<br>• Gross and fine motor function<br>• Problem-solving<br>• Personal-social behavior	• Children born to mothers who consumed >300 mg caffeine/day had 1.11-fold increased odds of gross motor developmental delay at 12 months of age<br>• Developmental delays in gross motor skills	• Caffeine exposure measurement errors<br>• Evaluations based on caregiver reports<br>• Influence of socioeconomic factors and maternal depressive tendencies
Gleason, J.L., et al. (2021) [2]	Cohort study	2,055	Plasma concentrations of caffeine and paraxanthine<br><br>Self-reported caffeinated beverage consumption	Nonsmoking pregnant women at 10-13 weeks of gestation with defined caffeine metabolism (CYP1A2*1F)		• Birth weight<br>• Length<br>• Head, abdominal, arm, and thigh circumferences<br>• Skin fold and fat mass measures	• Increasing caffeine and paraxanthine measures were significantly associated with lower birth weight, shorter length, and smaller head, arm, and thigh circumference	• Low correlation between self-reported intake and plasma caffeine/ paraxanthine levels<br>• Caffeine exposure measurement errors<br>• Differences in individual caffeine metabolism rates<br>• Missing data on the timing of last caffeine consumption

			measured/reported					• Single plasma caffeine/paraxanthine measurement during pregnancy
Gleason, J.L., et al. (2022) [3]	Secondary analysis of two cohort studies: ECHO-FGS and CPP	788	Concentrations of caffeine and paraxanthine quantified from plasma (ECHO-FGS) or serum (CPP) measured during 1st trimester of pregnancy	Children aged from 4 to 8 years old (ECHO-FGS)<br><br>Children through the age of 8 years (CPP)	8 years	• Prenatal caffeine exposure<br>• BMI<br>• Weight<br>• Height<br>• Fat mass index and percentage<br>• Obesity risk	• Children of women with low measured caffeine and paraxanthine during pregnancy were shorter than the children of women who consumed no caffeine during pregnancy<br>• Serum and plasma caffeine were inversely associated with child weight in the third quartile and quintile of caffeine consumption only<br>• No clear patterns of BMI z-scores, child fat mass index, or fat percentage changes	• Not sufficient information on maternal diet, nausea or vomiting during pregnancy, or paternal height<br>• Only one growth measurement<br>• Unclear how measured caffeine and paraxanthine reflect habitual caffeine consumption
Kobayashi, S., et al. (2019) [4]	Prospective birth cohort	94,876	Self-administered FFQs	Fetuses	At birth	• Prenatal caffeine exposure<br>• Birthweight<br>• SGA<br>• Preterm birth	• Caffeine intake of ≥86.4 mg/day during pregnancy resulted in a dose-dependent increase in the risks of SGA and reduced birthweight z-score<br>• Prenatal caffeine consumption was associated	• Caffeine exposure measurement errors<br>• Lack of information about nausea and vomiting-related changes in food preferences and diet during pregnancy<br>• Data confounding factors and birth outcomes were

							• with an increased risk of preterm birth	missing for over 10,000 fetuses
Kukkonen, A., et al. (2024) [5]	Prospective cohort study	7,944	160-item FFQ during the first and third trimester	Pregnant women		• Prenatal caffeine exposure<br>• Birthweight	• Moderate (51-200mg/day) and high (>200mg/day) caffeine intake during early pregnancy is associated with SGA<br>• Caffeine intake during early pregnancy is more significant for the development of SGA than caffeine intake later in pregnancy	• Caffeine exposure measurement errors
Haan, E., et. al. (2022) [6]	Prospective longitudinal cohort study	ALSPAC – 14,541<br>GenR – 9,778<br>MoBa – 284,900	Self-report questionaries	Pregnant women and their children	7-8 years	• Prenatal caffeine exposure<br>• ADHD symptoms in children	• No clear evidence of prenatal caffeine consumption and ADHD risk	• Caffeine exposure measurement errors<br>• Measurement errors in ADHD symptom assessment
Williford, E.M., et al. (2023) [7]	Secondary analysis of the NBDPS study (population-based case-control study)	30,285 cases and 11,502 control children	Computer-assisted telephone interviews	Liveborn, stillborn, or terminated pregnancies affected by one or more of 30 different categories of major structural		• Prenatal caffeine exposure<br>• Risk of 48 birth defects	• Relatively small increases in effect estimates for caffeine consumption and specific birth defects<br>• Did not find evidence of a dose-response pattern	• Caffeine exposure measurement errors<br>• Statistically significant results may be due to chance<br>• Small number of exposed cases, especially at the highest (≥300 mg/day) level of caffeine consumption

				defects (case children) Liveborn infants without major birth defects (control children)				• Potential residual confounding by maternal smoking
**Liver and Metabolic Dysfunctions in Children**								
[8] Voerman, E., et al.2020	Prospective population-based cohort study	4,770	Postal questionnaires	Pregnant women and their children	At the age of 10	• BMI<br>• Total fat index<br>• Android/gynoid fat mass ratio<br>• Overweight/obesity<br>• Abdominal or liver fat measure	• Children whose mothers consumed 4-5.9 (360-539 mg) and ≥6 units (540mg) of caffeine per day had a higher BMI, total body fat mass index, android/gynoid fat mass ratio, and abdominal subcutaneous and visceral fat mass indices<br>• Children whose mothers consumed 4-5.9 units (360-539 mg) of caffeine per day had a higher liver fat fraction	• Caffeine exposure measurement errors<br>• 39.6% of participants did not participate in the follow-up measurements at age 10<br>• Selection towards a more highly educated, healthier population<br>• maternal and child's physical activity and dietary habits
[9] Chen, L.-W., et al. 2019	Cross-Generation Cohort Study	558 mother-child pairs	Self-administered semiquantitative FFQ	Mother-child pairs	5- and 9-year follow-up	• Childhood obesity and adiposity<br>• Children's BMI and WC	• Maternal caffeine intake was associated with higher offspring BMI and WC z-scores<br>• Higher intakes of caffeine from coffee were associated	• Caffeine exposure measurement errors<br>• Shared familial environment between mothers and children

							with higher adiposity and obesity risk in offspring than tea caffeine<br>• A 100 mg/day increase in maternal caffeine intake was linked to a higher risk of overweight, obesity, and central obesity in offspring	• Included mothers were generally healthier<br>• Only early pregnancy caffeine intake data
[10] Zhao, Q., et al. 2021	Secondary analysis of the CANDLE study (prospective birth cohort study)	450 mother-child pairs	Self-administered questionnaires<br><br>Mothers' plasma samples collected during the second trimester of pregnancy	Mother-child pairs	From birth to the age of 4 (4 years)	• Children's BMI, body weight, and length	• Metabolic indicators of caffeine were positively associated with accelerated infant growth and childhood obesity	• Lack of replication samples<br>• Small sample size
**Cardiometabolic Complications**								
[11] Hinkle, S.N., et al. 2021	Secondary analysis of NICHD Fetal Growth Studies-Singletons	2,802	Self-reported<br><br>Plasma caffeine and paraxanthine were measured in	Pregnant women at 10-13 and 16- 22 gestational weeks		• GDM<br>• Preeclampsia<br>• GH<br>• Glucose concentrations<br>• Blood pressure	• Low and moderate caffeinated beverage intake (<200mg/day) early in the second trimester was associated with a lower risk for GDM, lower glucose levels at GDM screening, and	• The sample size was not large enough to separate analyses by beverage type<br>• Small number of women with pregnancy complications

	study (longitudinal pregnancy cohort study)		specimens collected at 10 to 13 weeks			• a more favorable cardiometabolic profile compared with no consumption<br>• Caffeine was not associated with gestational hypertension or preeclampsia	• Caffeine exposure measurement errors
[12] Gudeta, T.A., T.M. Regassa, and A.S. Belay 2019	Cross-sectional study	1,871	Pre-tested questionnaire	Pregnant women	• Anemia (hemoglobin level <11 g/dl during third trimester)	• Caffeine intake was associated with the development of anemia during pregnancy	• Caffeine exposure measurement errors
**Neurological Dysfunctions in Children**							
[13] Christensen, Z.P., E.G. Freedman, and J.J. Foxe 2021	Secondary analysis of the ABCD study	9,157	Parental reports	Children aged 9-10 years	• Prenatal caffeine exposure<br>• Brain structural outcomes at 27 major fiber tracts<br>• FA in the IFOF-LH and CST-LH<br>• Cognitive measures<br>• Psychopathology measures	• GCE was associated with poorer outcomes on all psychopathology measures<br>• GCE had a negligible effect on cognitive measures<br>• Higher FA values in both fiber tracts were associated with decreased neurodevelopmental problems and improved performance on	• Chose to focus on a single biomarker (mean FA)<br>• Approximate measure of task efficiency<br>• Measures did not isolate a particular pathology or cognitive domain<br>• Choice to iteratively refine the random effects parameter

							cognitive tasks<br>• Decreased association between FA in the CST-LH and task efficiency in the GCE group	
[14] Zhang, R., P. Manza, and N.D. Volkow 2021	Secondary analysis of the ABCD study	9,978	The Developmental History Questionnaire through parents' retrospective report	Children aged 9–11 years		• Prenatal caffeine exposure<br>• Weeks of prematurity<br>• Body weight at birth<br>• Puberty status<br>• BMI<br>• Waist circumference<br>• Sleep problems<br>• Psychopathology and behavior problems<br>• Cognition	• Altered brain structure<br>• Overlimit prenatal caffeine exposure*: higher BMI and greater soda consumption<br>• Daily prenatal caffeine: greater externalizing but not internalizing problems, greater somatic, oppositional defiant, and conduct problems<br>• Did not exhibit any significant cognitive differences nor sleep abnormalities	• No available information on maternal BMI, weight, or maternal weight gain during pregnancy<br>• Mothers who had greater behavioral, emotional, and social problems had higher caffeine intake during pregnancy<br>• Cross-sectional design<br>• Caffeine exposure measurement errors<br>• Small effect sizes<br>• Limited brain imaging analysis
**Risk of Diabetes in Mothers**								
[15] Kukkonen, A., et al. 2024	Prospective cohort study	2,214	160-item FFQ during first and third trimester (up to gestation week 13)	Pregnant women	Year-round	• GDM	• Moderate coffee intake during the first trimester of pregnancy was not associated with the risk of GDM, although the age-adjusted findings suggested a protective association	• Caffeine exposure measurement errors<br>• Potential confounding factors like smoking<br>• Coffee intake was higher later in pregnancy

							• Women who consumed cola drinks more than the median (33.3 mL/d) had an increased risk of GDM	
**Gut Microbiome**								
[16] Laue, H.E., et al. 2022	Prospective birth cohort study	365	Extracted from meconium passed after birth	Infants and children aged 6-7 years	Recruited in early pregnancy or at the time of birth<br><br>Follow-up at 6-7 years old	• Prenatal caffeine exposure<br>• Gut microbiome diversity	• Prenatal caffeine exposure was not associated with differences in the microbiome	• Small sample size<br>• Exposure assessment assay only quantified concentrations of the parent compounds and not their metabolites
**Risk of Childhood Neoplasms**								
[17] Karalexi et. al. 2019	Pooled case-control study	554 (AML) 1419 (controls)	Self-administered food frequency or general questionnaires	Children aged 0-14 with AML		• Prenatal coffee and tea exposure<br>• Risk of childhood AML development<br>• Hyperdiploidy	• Minor evidence for the correlation of prenatal maternal coffee consumption and childhood AML development, particularly those drinking >1 cup of coffee per day<br>• No associations between maternal tea consumption and risk of childhood AML<br>• Inverse association between hyperdiploidy and tea consumption	• Caffeine exposure measurement errors<br>• Small respective subgroups of AML cases<br>• Residual confounding
**Preterm Birth and Pregnancy Loss**								

[18] Manuck, T.A., et al. 2019	Case-control analysis from a prospective cohort study	42	Plasma blood sample drawn at <28 weeks of gestation	Pregnant women with a high risk of spontaneous preterm birth	• Levels of endogenous metabolites and exogenous exposure in maternal plasma<br>• Preterm birth	• Increased level of caffeine in blood plasma was associated with preterm birth	• Standard levels for the majority of the metabolites studied have not been established<br>• Small sample size<br>• Metabolites measured at a single time point
**Reproductive Health of Children**							
[19] Eubanks, A.A., et al, 2020	Secondary analysis of the EAGeR trial	1,228	Questionnaires	Women exposed to caffeine in utero	• Prenatal caffeine exposure<br>• AMH concentration in serum	• Prenatal caffeine exposure was associated with lower AMH in female offspring	• Relies on the participants' recall and knowledge of their mother's health during their in-utero development<br>• AMH values were selected based on studies of infertile women<br>• Limited ability to evaluate a dose-response or threshold effects ("yes" and "no" questions only)<br>• The utility of AMH as a marker for fertility is still under debate<br>• Selection bias
**B.META-ANALYSES**							
**Pregnancy Loss**							
[20]	Meta-analysis of	2 studies regarding caffeine		Pregnant women	• Caffeine consumption during pregnancy<br>• Risk of RLP	• No increased risk of RPL in the general population of women with a higher level of	• Diversity in the methodology and methods of reporting RPL

Ng, K.Y.B., et al. 2021	observational studies	exposure and the risk of RLP (n=1,417)				caffeine intake compared to lower intake	• Limited to studies in English<br>• The data captured by studies in this review relate to the lifestyle reported at the time of the study
**Labor Dystocia**							
[21] Jochumsen, S., et al. 2023	Meta-analysis of cohort studies	7 studies, of which one regarding caffeine exposure (n=2,810)	Self-administered questionnaire	Low-risk nulliparous women	• Risk of labor dystocia	• Caffeine intake of 200–299 mg during pregnancy was associated with a higher frequency of labor dystocia compared to women who reported a caffeine intake of 0–99 mg per day	• Diagnostic criteria for labor dystocia are not clearly defined<br>• Most studies did not clarify at which stage of labor the complication was examined
**DNA Methylation**							
[22] Schellhas, L., et al. 2023	Meta-analysis of prospective pregnancy and birth cohorts	6 studies (n=3,725)	Self-reported questionnaires	Pregnant women	• Intrauterine caffeine exposure<br>• Offspring DNA methylation in cord blood	• No support for an intrauterine effect of caffeine on DNA methylation at birth	• Caffeine exposure measurement errors<br>• Only assessed at regions available on the 450k array, which only covers around 2% of CpG sites of the entire epigenome<br>• Only assessed offspring DNAm in blood<br>• Potential confounding factors

								• A bigger sample size may be required
**Neurological and Neurodevelopmental Disorders in Children**								
[23]<br>Hu, Z., et al. 2024	Meta-analysis of case-control studies	5 studies	Interviews, Questionnaires,<br>FFQ	Children		• Risk of CBTs	• Increased risk of CBTs was associated with maternal consumption of caffeinated beverages during pregnancy, especially coffee<br>• No significant association between tea consumption during pregnancy and the risk of CBTs	• Most of the involved studies were case-control studies<br>• Caffeine exposure measurement errors
[24]<br>Patti, M.A., et al. 2019	Meta-analysis of prospective cohort studies	2 studies (n=389)	Study-specific questionnaires (twice during pregnancy)	Children aged 3-8 years		• ASD-related behaviors using SRS<br>• Prenatal caffeine exposure	• Maternal caffeine intake during pregnancy was associated with modest increases in ASD-related behaviors	• Caffeine exposure measurement errors<br>• Differences in the features of participants<br>• SRS is not a clinical diagnosis of ASD<br>• Potential residual confounding<br>• Selection bias
[25]<br>Haan, E., et al. 2022	Meta-analysis of two longitudinal cohort studies, one case-control	3 studies on ADHD (n = 3,627–24,156)	Self-reports	Children		• Prenatal caffeine exposure<br>• ADHD<br>• ODD	• No evidence for an association was observed between maternal prenatal caffeine consumption and offspring ADHD<br>• A study on ODD rated as a very high risk of bias found	• Excluded studies reporting on symptom scores or other continuous scales<br>• Low number of studies<br>• Included only English language studies

	study, and one cross-sectional study	One study on ODD (n = 5,924)					weak evidence of an association in girls	• Caffeine exposure measurement errors<br>• Studies varied greatly in age when ADHD and ODD were assessed
**Developmental Delays and Abnormalities in Children**								
[26] Brito Nunes, C., et al. 2023	Used summary results data from the GWAS	UKBB (n=193,948) ALSPAC (n=14,541)	Self-reported questionnaires	Pregnant women		• Birthweight<br>• Number of stillbirths<br>• Number of miscarriages<br>• Gestational age<br>• Pre-term birth	• No change in risk of sporadic miscarriages, stillbirths, pre-term birth, or effect on gestational age<br>• Association between increased coffee consumption and higher birthweight, the magnitude of the effect was inconsistent	• SNPs used in the analysis were identified in the general population rather than in pregnant women<br>• Caffeine exposure measurement errors
[27] Soltani, S., et al. 2023	Meta-analysis of cohort studies	7 studies (n=29,168)	Questionnaires	Pregnant women	-	• Risk of LBW	• Positive association between maternal caffeine intake and risk of LBW<br>• Each additional 100 mg/day of maternal caffeine intake was significantly associated with an increased risk of LBW	• Inaccuracies in data extraction<br>• Based on observational studies<br>• Caffeine exposure measurement errors<br>• Conducted on relatively healthy pregnant women with singleton pregnancies<br>• Publication bias
[28] Jin, F. and C.	Meta-analysis of	15 studies	Questionnaires, Interviews, Surveys	Pregnant women		• Risk of LBW<br>• Risk of childhood overweight	• Maternal caffeine intake during pregnancy is associated with a higher risk	• Observational design<br>• Significant heterogeneity among studies results

Qiao, 2021	cohort studies	(n=102,347)				• Risk of childhood obesity	of LBW and childhood overweight and obesity	• The size of the study population, the length of cohort follow-up, and the adjustments for confounders varied from study to study
**Cardiometabolic complications**								
[29] Chen, B., et al. 2022	Meta-analysis of observational studies	10 studies (n=114,984)	Questionnaires, Interviews, FFQ	Pregnant women		• GH<br>• Preeclampsia	• Caffeine exposure during pregnancy was not significantly associated with the risk of GH/preeclampsia	• Limited number of articles<br>• Caffeine exposure measurement errors<br>• Only observational studies included<br>• Some studies did not control for covariates or ignored some potential residual confusion<br>• Exclusion of non-English articles

*Daily recommended caffeine intake in pregnancy by ACOG: <200mg

ABCD—Adolescent Brain and Cognitive Development, ACOG—American College of Obstetricians and Gynecologists, ADHD—Attention-deficit hyperactivity disorder, ALL—Acute lymphoblastic leukemia, ALSPAC—Avon Longitudinal Study of Parents and Children, AMH—Anti-müllerian hormone, AML—Acute Myeloid Leukemia, ASD—Autism spectrum disorder, BMI—Body mass index, CANDLE—Conditions Affecting Neurocognitive Development and Learning in Early Childhood, CBT—Childhood brain tumors, CpG—Cytosine-phosphate-Guanine , CPP—The Collaborative Perinatal Project, CST-LH—Corticospinal tract of the left hemisphere, CYP1A2*F—Cytochrome P450 family 1 subfamily A polypeptide 2 Variant 1F, DNA—Deoxyribonucleic Acid, DNAm—DNA methylation, EAGeR—The Effects of Aspirin in Gestation and Reproduction (multi-center, double-blind, block-randomized, placebo-controlled trial evaluating the effects of low dose aspirin on live birth), ECHO-FGS—The Environmental Influences on Child Health Outcomes cohort of the National Institute of Child Health and Human Development Fetal Growth Studies, FA—Fractional anisotropy, FFQ—The Food Frequency Questionnaire, GCE—Gestational caffeine exposure, GDM—Gestational diabetes mellitus, GenR—Generation R study, GH—Gestational hypertension, GWAS—Genome-wide association study, IFOF-LH—Inferior fronto-occipito fasciculus of the left

hemisphere, LBW—Low birth weight <2500 g, MoBa—Mother, Father and Child Cohort study, NBDPS—National Birth Defects Prevention Study, NICHD—National Institute of Child Health and Human Development, ODD—Oppositional defiant disorder, RLP—Recurrent pregnancy loss, SGA—Small-for-gestational-age, SNPs—single-nucleotide polymorphisms, SRS—Social Responsiveness Scale, UKBB—UK Biobank, WC—Waist circumference

1. Nishihara, S., et al., *Association between maternal caffeine intake during pregnancy and child development at 6 and 12 months: The Japan Environment and Children's Study.* Early Human Development, 2022. **171**: p. 105607.
2. Gleason, J.L., et al., *Association Between Maternal Caffeine Consumption and Metabolism and Neonatal Anthropometry.* JAMA Network Open, 2021. **4**(3): p. e213238.
3. Gleason, J.L., et al., *Association of Maternal Caffeine Consumption During Pregnancy With Child Growth.* JAMA Network Open, 2022. **5**(10): p. e2239609.
4. Kobayashi, S., et al., *Dose-dependent associations between prenatal caffeine consumption and small for gestational age, preterm birth, and reduced birthweight in the Japan Environment and Children's Study.* Paediatric and Perinatal Epidemiology, 2019. **33**(3): p. 185-194.
5. Kukkonen, A., et al., *Maternal caffeine intake during pregnancy and the risk of delivering a small for gestational age baby: Kuopio Birth Cohort.* Archives of Gynecology and Obstetrics, 2024. **310**(1): p. 359-368.
6. Haan, E., et al., *Prenatal smoking, alcohol and caffeine exposure and maternal-reported attention deficit hyperactivity disorder symptoms in childhood: triangulation of evidence using negative control and polygenic risk score analyses.* Addiction, 2022. **117**(5): p. 1458-1471.
7. Williford, E.M., et al., *Maternal dietary caffeine consumption and risk of birth defects in the <scp>National Birth Defects Prevention Study</scp>, 1997–2011.* Birth Defects Research, 2023. **115**(9): p. 921-932.
8. Voerman, E., et al., *Associations of maternal caffeine intake during pregnancy with abdominal and liver fat deposition in childhood.* Pediatric Obesity, 2020. **15**(5).
9. Chen, L.-W., et al., *Maternal, but not paternal or grandparental, caffeine intake is associated with childhood obesity and adiposity: The Lifeways Cross-Generation Cohort Study.* The American Journal of Clinical Nutrition, 2019. **109**(6): p. 1648-1655.
10. Zhao, Q., et al., *Associations of prenatal metabolomics profiles with early childhood growth trajectories and obesity risk in African Americans: the CANDLE study.* International Journal of Obesity, 2021. **45**(7): p. 1439-1447.
11. Hinkle, S.N., et al., *Assessment of Caffeine Consumption and Maternal Cardiometabolic Pregnancy Complications.* JAMA Network Open, 2021. **4**(11): p. e2133401.
12. Gudeta, T.A., T.M. Regassa, and A.S. Belay, *Magnitude and factors associated with anemia among pregnant women attending antenatal care in Bench Maji, Keffa and Sheka zones of public hospitals, Southwest, Ethiopia, 2018: A cross -sectional study.* PLOS ONE, 2019. **14**(11): p. e0225148.
13. Christensen, Z.P., E.G. Freedman, and J.J. Foxe, *Caffeine exposure in utero is associated with structural brain alterations and deleterious neurocognitive outcomes in 9–10 year old children.* Neuropharmacology, 2021. **186**: p. 108479.
14. Zhang, R., P. Manza, and N.D. Volkow, *Prenatal caffeine exposure: association with neurodevelopmental outcomes in 9- to 11-year-old children.* Journal of Child Psychology and Psychiatry, 2022. **63**(5): p. 563-578.

15. Kukkonen, A., et al., *Maternal caffeine, coffee and cola drink intake and the risk of gestational diabetes – Kuopio Birth Cohort.* Primary Care Diabetes, 2024. **18**(3): p. 362-367.
16. Laue, H.E., et al., *In Utero Exposure to Caffeine and Acetaminophen, the Gut Microbiome, and Neurodevelopmental Outcomes: A Prospective Birth Cohort Study.* International Journal of Environmental Research and Public Health, 2022. **19**(15): p. 9357.
17. Karalexi, M.A., et al., *Coffee and tea consumption during pregnancy and risk of childhood acute myeloid leukemia: A Childhood Leukemia International Consortium (CLIC) study.* Cancer Epidemiology, 2019. **62**: p. 101581.
18. Manuck, T.A., et al., *Metabolites from midtrimester plasma of pregnant patients at high risk for preterm birth.* American Journal of Obstetrics & Gynecology MFM, 2021. **3**(4): p. 100393.
19. Eubanks, A.A., et al., *Recalled maternal lifestyle behaviors associated with anti-müllerian hormone of adult female offspring.* Reproductive Toxicology, 2020. **98**: p. 75-81.
20. Ng, K.Y.B., et al., *Systematic review and meta-analysis of female lifestyle factors and risk of recurrent pregnancy loss.* Scientific Reports, 2021. **11**(1).
21. Jochumsen, S., et al., *Maternal factors associated with labor dystocia in low-risk nulliparous women. A systematic review and meta-analysis.* Sexual & Reproductive Healthcare, 2023. **36**: p. 100855.
22. Schellhas, L., et al., *Maternal Caffeine Consumption During Pregnancy and Offspring Cord Blood DNA Methylation: An Epigenome-Wide Association Study Meta-Analysis.* Epigenomics, 2023. **15**(22): p. 1179-1193.
23. Hu, Z., et al., *Maternal smoking, consumption of alcohol, and caffeinated beverages during pregnancy and the risk of childhood brain tumors: a meta-analysis of observational studies.* BMC Public Health, 2024. **24**(1).
24. Patti, M.A., et al., *Association between self-reported caffeine intake during pregnancy and social responsiveness scores in childhood: The EARLI and HOME studies.* PLOS ONE, 2021. **16**(1): p. e0245079.
25. Haan, E., et al., *Prenatal smoking, alcohol and caffeine exposure and offspring externalizing disorders: a systematic review and meta-analysis.* Addiction, 2022. **117**(10): p. 2602-2613.
26. Brito Nunes, C., et al., *Mendelian randomization study of maternal coffee consumption and its influence on birthweight, stillbirth, miscarriage, gestational age and pre-term birth.* International Journal of Epidemiology, 2023. **52**(1): p. 165-177.
27. Soltani, S., et al., *Maternal caffeine consumption during pregnancy and risk of low birth weight: a dose–response meta-analysis of cohort studies.* Critical Reviews in Food Science and Nutrition, 2023. **63**(2): p. 224-233.
28. Jin, F. and C. Qiao, *Association of maternal caffeine intake during pregnancy with low birth weight, childhood overweight, and obesity: a meta-analysis of cohort studies.* International Journal of Obesity, 2021. **45**(2): p. 279-287.
29. Chen, B., et al., *The association between caffeine exposure during pregnancy and risk of gestational hypertension/preeclampsia: A meta-analysis and systematical review.* Journal of Obstetrics and Gynaecology Research, 2022. **48**(12): p. 3045-3055.
